# Persistent Release of IL-1s from Skin Is Associated with Systemic Cardio-Vascular Disease, Emaciation and Systemic Amyloidosis: The Potential of Anti-IL-1 Therapy for Systemic Inflammatory Diseases

**DOI:** 10.1371/journal.pone.0104479

**Published:** 2014-08-13

**Authors:** Keiichi Yamanaka, Takehisa Nakanishi, Hiromitsu Saito, Junko Maruyama, Kenichi Isoda, Ayumu Yokochi, Kyoko Imanaka-Yoshida, Kenshiro Tsuda, Masato Kakeda, Ryuji Okamoto, Satoshi Fujita, Yoichiro Iwakura, Noboru Suzuki, Masaaki Ito, Kazuo Maruyama, Esteban C. Gabazza, Toshimichi Yoshida, Motomu Shimaoka, Hitoshi Mizutani

**Affiliations:** 1 Department of Dermatology, Mie University, Graduate School of Medicine, Tsu, Mie, Japan; 2 Department of Animal Genomics, Functional Genomics Institute, Mie University Life Science Research Center, Tsu, Mie, Japan; 3 Department of Clinical Engineering, Suzuka University of Medical Science, Suzuka, Mie, Japan; 4 Anesthesiology and Critical Care Medicine, Mie University, Graduate School of Medicine, Tsu, Mie, Japan; 5 Pathology and Matrix Biology, Mie University, Graduate School of Medicine, Tsu, Mie, Japan; 6 Mie University Research Center for Matrix Biology, Tsu, Mie, Japan; 7 Cardiology, Mie University, Graduate School of Medicine, Tsu, Mie, Japan; 8 Division of Experimental Animal Immunology, Tokyo University of Science, Noda, Chiba, Japan; 9 Immunology, Mie University, Graduate School of Medicine, Tsu, Mie, Japan; 10 Molecular Pathology and Cell Adhesion Biology, Mie University, Graduate School of Medicine, Tsu, Mie, Japan; Keio University School of Medicine, Japan

## Abstract

The skin is an immune organ that contains innate and acquired immune systems and thus is able to respond to exogenous stimuli producing large amount of proinflammatory cytokines including IL-1 and IL-1 family members. The role of the epidermal IL-1 is not limited to initiation of local inflammatory responses, but also to induction of systemic inflammation. However, association of persistent release of IL-1 family members from severe skin inflammatory diseases such as psoriasis, epidermolysis bullosa, atopic dermatitis, blistering diseases and desmoglein-1 deficiency syndrome with diseases in systemic organs have not been so far assessed. Here, we showed the occurrence of severe systemic cardiovascular diseases and metabolic abnormalities including aberrant vascular wall remodeling with aortic stenosis, cardiomegaly, impaired limb and tail circulation, fatty tissue loss and systemic amyloid deposition in multiple organs with liver and kidney dysfunction in mouse models with severe dermatitis caused by persistent release of IL-1s from the skin. These morbid conditions were ameliorated by simultaneous administration of anti-IL-1α and IL-1β antibodies. These findings may explain the morbid association of arteriosclerosis, heart involvement, amyloidosis and cachexia in severe systemic skin diseases and systemic autoinflammatory diseases, and support the value of anti-IL-1 therapy for systemic inflammatory diseases.

## Introduction

Cardiovascular diseases, obesity, liver and renal diseases are going to be the major pathologies of the 21th century. A significant interaction between systemic inflammatory changes and systemic organ disease during the metabolic syndromes has been reported. Skin is a prototype of immune system that can respond to exogenous stimuli triggering systemic inflammation by promoting the migration of bone-derived hematopoietic cells. Cardiovascular and other systemic disorders have been reported in severe systemic skin diseases including psoriasis, epidermolysis bullosa (EB), hidradenitis suppurativa, atopic dermatitis (AD) and desmoglein-1 deficiency [1]–[4]. However, the mechanistic pathways of systemic organ involvement during inflammatory skin diseases are unclear.

The role of epidermal keratinocytes is to trigger local and systemic inflammation by releasing stored IL-1s leading to activation of the immune system and the cytokine cascade. Skin scratching, cracking by xerosis and dermatitis promote the release of active IL-1α through a calcium-activated protease calpain [5] and/or CTL/NK protease granzyme B mechanism [6]. IL-1β is stored as an inactive precursor and can be activated by specific enzymes (e.g. caspase-1/IL-1β converting enzyme) before being secreted. IL-1 plays a key role in allergic dermatitis [7].

Chronic inflammation can cause aberrant remodeling of vascular and fatty tissues, potentially resulting in atherosclerosis and obesity/lipodystrophy [8]. Anti-inflammatory agents have been used as a novel therapeutic approach to reverse these pathological conditions [9]; for example, clinical trials using inhibitors of IL-1 have been carried out to treat atherosclerosis [10]. IL-1 is believed to affect primarily surrounding cells at sites of tissue injury. Bone marrow-derived hematologic cells (e.g., monocytes/macrophages) migrate into vascular walls where they secrete IL-1 that can stimulate resident cells (e.g. vascular smooth muscle cells, endothelial cells), and thereby contribute to the pathogenesis of atherosclerosis [11]. In addition to its primary role as a local mediator, excessive expression of IL-1 can spill over into the systemic circulation and affect remote organs. Sustained skin inflammation in severe epidermal inflammation patients including psoriasis, EB, AD can lead to aberrant secretion of IL-1, which can potentially cause vascular and visceral pathologies. The pathological effects of hypercytokinemia have been well documented in some cases of acute and usually self-limiting inflammation, typically caused by infections (e.g., cytokine storm in severe influenza virus infection-associated acute respiratory distress syndrome) [12] as well as in cases of cancer-associated chronic inflammation leading to cachexia [13]. However, the exact morbid conditions induced by high systemic levels of IL-1 during severe diseases with persistent and intensive epidermis injury remains largely unknown.

We addressed this problem by using keratin-14 driven caspase-1 transgenic mice (KCASP1Tg) [14] and a keratinocyte-specific mature IL-18-transgenic mice line (KIL-18Tg) that we have previously developed [7]. Here, we show that KCASP1Tg and KIL-18Tg mice with dermatitis have severe pathology in systemic organs other than the skin including aberrant remodeling of fatty and connective tissues, and extensive amyloid deposition with organ dysfunction, and that these abnormalities improved with the use of anti IL-1α/β antibodies.

## Materials and Methods

### Transgenic mice

Transgenic mice in which keratinocytes specifically overexpress the human caspase-1 gene with the K14 promoter, designated as KCASP1Tg, were used in this study [14]. A keratinocyte-specific mature IL-18-transgenic mice line (KIL-18Tg) that has been previously characterized was also used [7]. C57BL/6 littermate mice were used as controls. We closely monitored these mice until they were 6-months old. KIL-18Tg mice of less than 1-year old showed no findings of dermatitis; these mice are referred as KIL-18Tg(−). After 1-year old, the KIL-18Tg mice develop chronic dermatitis; these mice are referred as KIL-18Tg(+). Animal care was performed according to current ethical guidelines, and the experimental protocol was approved by the Mie University Board Committee for Animal Care and Use (#22-39).

### Percentage of skin alteration and measurement of body weight

Skin alterations and body weight were observed at two-week intervals. Skin lesions and total body surface area were evaluated by marking on lucent plastic film, and then expressed as the percentage of area versus the total-body surface (n = 10, each group). Long-term observation was also performed in KIL-18Tg mice (n = 7, each group).

### Computed tomography and 3-dimensional analysis

Under total anesthesia using isoflurane inhalation (Abbott, Abbott Park, IL), mice underwent micro X-ray CT angiography (Rigaku, Tokyo, Japan), and the data was analyzed using i-VIEW-R software (Rigaku). Body fat percentage was calculated and 3-dimensional graphics were obtained (n = 6, each group).

### Plasma cytokine and cholesterol concentration

Plasma samples were collected from 6-months old mice (n = 10, each group) and eighteen-months old KIL-18Tg(+) mice (n = 7). Plasma cytokine levels were measured by specific ELISA kits (IL-1α, β: R&D systems, Minneapolis, MN, USA, IL-18: MBL, Nagoya, Japan) according to the manufacturer's instructions. TG, HDL, LDL cholesterol levels, liver and renal functions were measured with commercially available systems. Serum leptin (R&D systems), adiponectin (Otsuka, Tokyo, Japan) and amyloid A protein levels (Life Technologies, Carlsbad, CA) were measured by specific ELISA following the manufacturer's instructions.

### IL-1 neutralization in KCASP1Tg mice

Ten µg of anti-IL-1α, β and α plus β neutralizing antibodies (BioLegend, CA, USA) were injected intraperitoneally into KCASP1Tg mice once-a-week from 1-month to 6-months old. PBS-treated KCASP1Tg littermates were used as controls (n = 7, each group).

### Intra-peritoneal injection of recombinant protein

Intra-peritoneal injection of 1 µg of recombinant protein (IL-1α or IL-1 β (BioLegend)) into normal mice was performed 3 times/week during the period the mice were 6 to 16 weeks old, and physical changes were compared with normal PBS-treated mice (n = 6, each group).

### Histological analysis

Abdominal adipose tissue and aorta specimens were fixed in 10% buffered neutral formaldehyde and embedded in paraffin. Histological sections were 6-µm thick and stained with hematoxylin and eosin (H&E). The aorta sections were also stained with Elastica van Gieson stain (EVG). The sections for liver, kidney, and spleen were stained with H&E and with Congo-red (n = 7, each group).

### Culture of adipocytes with conditioned medium from skin culture

Skin culture was prepared by taking 1 cm^2^ skin sections from 6-months-old normal or KCASP1Tg mice, minced with scissors, and harvested in 2 mL of RPMI 1640 containing 10% FCS, 2 mM L-glutamine, 100 U/ml penicillin, and 100 µg/ml streptomycin plated in a 24-well culture plate (Coster, NY, USA). In some experiments the skin post culture medium was treated with 1 µg of anti-IL-1α, anti-IL-1β, or a mixture of anti IL-1α and β neutralizing antibodies (BioLegend), and then incubated for 2 hours. The mouse embryonic fibroblast adipose-like cell line 3T3-L1 (ATCC, Manassas, VA) was cultured as previously reported for 10 days [15], and then differentiated into mature adipocytes. The medium was changed using the following supplementation: skin culture supernatant derived from normal mice, KCASP1Tg, anti-IL-1α and/or anti-IL-1β neutralizing antibodies-treated KCASP1Tg skin supernatant. On day 14, cultured cells were rinsed with PBS, stained with oil red O (Sigma-Aldrich, St. Louis, MO) and haematoxylin, and then observed under microscope (n = 7, each group).

### Cytokine measurement in skin culture supernatant

IL-1α and IL-β levels were measured in skin culture supernatant with FlowCytomix (eBioscience) following the manufacturer's instructions (n = 7, each group).

### Snapping tension of abdominal aorta

Mice were anesthetized with sodium pentobarbital (50 mg/kg, i.p.), and the abdominal arteries were isolated, gently cleaned of fat and connective tissue, and cut into rings (1 mm in length) as previously reported [16]. Rings were suspended vertically between stainless steel hooks in organ baths (20 ml) with modified Krebs–Henseleit solution (room temperature) containing (in mM): NaCl 115, KCl 4.7, CaCl2 2.5, MgCl2 1.2, NaHCO3 25, KH2PO4 1.2, and dextrose 10 in order to record the tension. The changes in isometric tension were measured with a force–displacement transducer (TB-651T; Nihon Kohden, Tokyo, Japan) connected to a carrier amplifier (EF601G; Nihon Kohden) and recorded with a pen recorder (WT-645G; Nihon Kohden). The bath medium was maintained at 37°C and bubbled continuously with 95% air and 5% CO2. Arterial rings were washed and allowed to equilibrate for 30 min. To measure the tension when the aortic ring snapped, the isometric tension was gradually increased (n = 12, each group).

### Peripheral blood pressure and thermography

Blood pressure was measured when mice were 6 months old using the BP-98A tail cuff system (Softron, Tokyo, Japan). This was done while the animals were still conscious as previously described [17] [18], and the peripheral blood circulation was measured by thermography (FLIR systems, Boston, MA) (n = 10, each group). These measurements were also performed in eighteen-months old KIL-18Tg(+) mice (n = 7).

### Statistical analysis

Statistical analysis was performed using the Friedman test. Student t test was used for the analysis of snapping tension results. P<0.05 was considered as significant.

## Results

### KCASP1Tg and KIL-18Tg mice with dermatitis show emaciation and altered lipid metabolism

**Figure 1:**
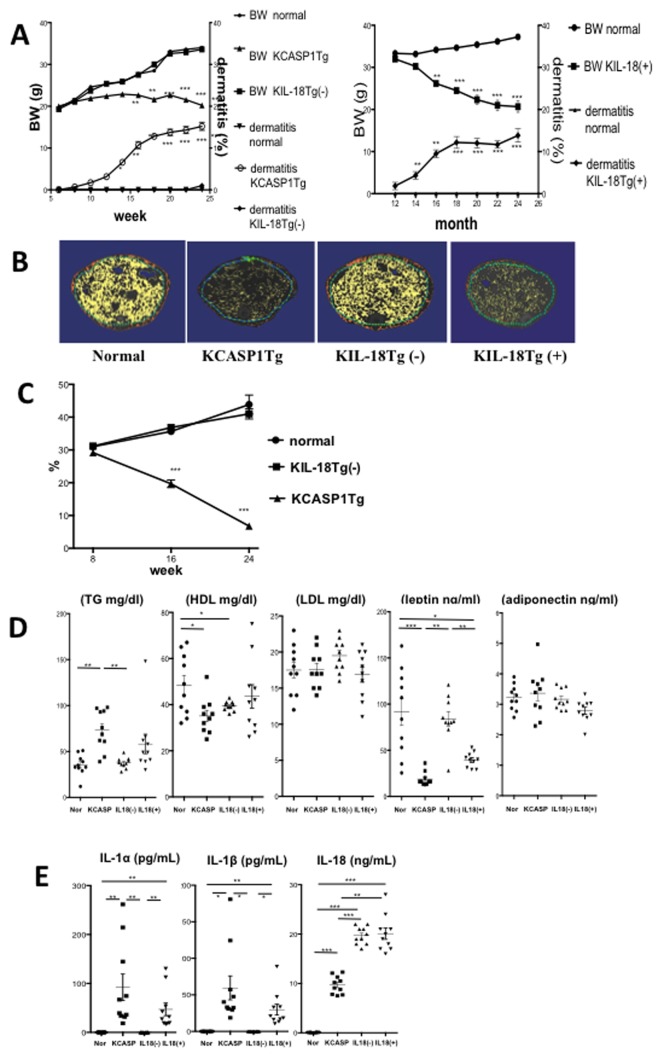
KCASP1Tg and KIL-18Tg(+) mice showed emaciation and altered lipid metabolism in addition to dermatitis. **A**) KCASP1Tg mice had erosive dermatitis at week 8, which spread across the entire face and trunk when mice were 5-months old. KIL-18Tg mice showed no dermatitis at 6 months of age, KIL-18Tg(−). Weight loss began at 10 weeks in KCASP1Tg mice, but not in age matched KIL-18Tg mice (n = 10, each group). The left Y-axis shows body weight and the right Y-axis shows the percentage of dermatitis. KIL-18Tg mice developed dermatitis at 1-year old, followed by weight loss, KIL-18Tg(+) (n = 7, each group) (*p<0.05, **p<0.001, ***p<0.0001). **B**) CT scan of KCASP1Tg mice at 6 months of age revealed a dramatic decrease in visceral fat as shown in yellow compared to normal control or KIL-18Tg(−) mice. Eighteen-year old KIL-18Tg(+) showed decreased visceral fat. Subcutaneous fat (in orange color) was also decreased in KCASP1Tg and KIL-18Tg(+). **C**) A comparison of the somatic fat ratio across the three groups determined by CT scan at 2, 4 and 6 months of age, and a decrease was observed in KCASP1Tg mice compared to the other two groups (n = 6, each group). **D**) Six-month-old KCASP1Tg mice showed decreased plasma HDL cholesterol and leptin levels, as well as increased triglyceride levels. LDL cholesterol and adiponectin levels remained normal. Eighteen-months old KIL-18Tg(+) mice showed decreased leptin levels. No significant change was identified in the triglyceride, HDL and LDL cholesterol, and adiponectin levels in KIL-18Tg(+) mice. **E**) Plasma IL-1α and β levels were elevated in 6-months old KCASP1Tg mice. IL-1 levels were under the detection limit in KIL-18Tg(−) mice, but were elevated in 18-months old KIL-18Tg(+) mice. Plasma IL-18 levels were increased in both KCASP1Tg and KIL-18Tg mice (n = 10).

The dermatitis in KCASP1Tg mice gradually spreads across the entire face and trunk, covering approximately 15% of the body surface when the mouse was 5-months old. Notably, KCASP1Tg mice gradually became emaciated as the dermatitis spreads. In these mice, weight loss began at week 10 (Fig. 1A). CT scans revealed a dramatic decrease in somatic and subcutaneous fat tissues (Fig. 1B,C). Caspase-1 is the converting enzyme for immature IL-1β and IL-18, therefore to rule out the possibility that this emaciation was primarily induced by high circulating level of IL-18, further studies were performed using the IL-18 transgenic mice (KIL-18Tg)[7]. KIL-18Tg of less than one-year of age did not show any phenotype; these mice are referred as KIL-18Tg(-) (Fig. 1A). After becoming 1-year old, the KIL-18Tg mice develop dermatitis followed by weight loss; these mice are referred as KIL-18Tg(+) (Fig. 1A,B). Six-months old KCASP1Tg mice showed decreased plasma levels of HDL cholesterol and leptin but elevated plasma level of triglycerides (Fig. 1D). Eighteen-months old KIL-18Tg(+) mice showed decreased plasma level of leptin but no significant changes were observed in the levels of triglyceride, HDL and LDL cholesterol, and adiponectin.

In addition, KCASP1Tg mice have increased levels of IL-1α, IL-1β, and IL-18 (Fig. 1E); this increase in plasma levels was due to leakage of IL-1α from injured keratinocytes and to conversion of the precursors of IL-1β and IL-18 by the transgene encoded caspase-1. The plasma concentration of IL-18 was already elevated in KIL-18Tg(−), but the IL-1 level was under the detection limit. Both IL-1α and IL-1β were dramatically increased in KIL-18Tg(+) mice after onset of dermatitis. We postulate that IL-1α in KIL-18Tg(+) mice is released from injured keratinocytes, and that pro-IL-1β is converted by endogenous caspase-1 before its release. These data suggest that skin derived IL-1α and IL-1β rather than IL-18 are the primary cause of emaciation.

### IL-1α and IL-1β are the primary cause of fat tissue remodeling

**Figure 2:**
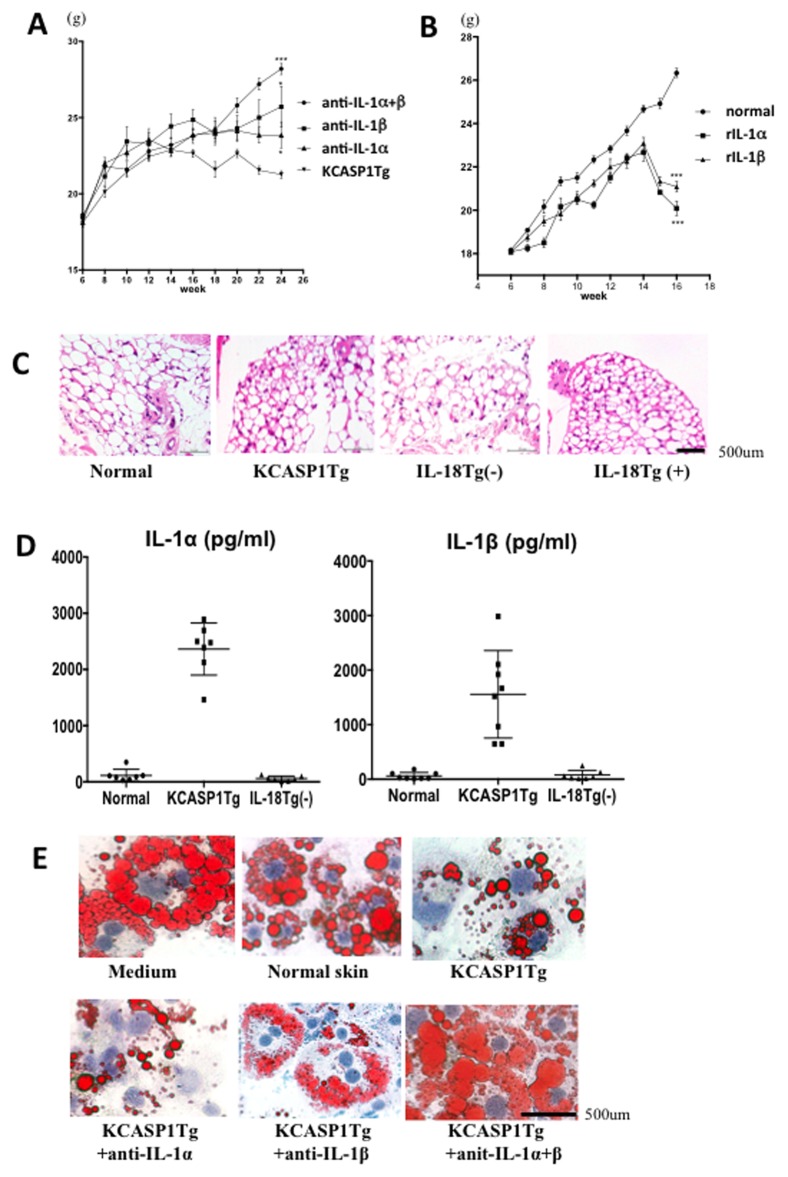
IL-1α and IL-1β trigger fat tissue remodeling. **A**) KCASP1Tg mice were treated once-a-week with an intra-peritoneal injection of 10 µg anti-IL-1α and/or IL-1β neutralizing antibodies between 4 to 24 weeks of age. PBS-treated KCASP1Tg littermates were used as controls. The body weight loss was ameliorated by either anti-IL-1α, anti-IL-1β or anti-IL-1α/β administration, (n = 7, each group). **B**) Emaciation was reproduced by administering 1 µg of recombinant IL-1α or IL-1β protein 3 times per week from 6 to 16 weeks of age to wild type mice compared to PBS-injected mouse controls from 6 to 16 weeks (n = 6, each group). **C**) H&E staining of abdominal adipose tissue revealed that the adipocytes were large and plump in shape in normal control and 6-months old KIL-18Tg(−) mice; they were small and round, however, in 6-month-old KCASP1Tg and 18-months old KIL-18Tg(+) mice. The number of infiltrating mononuclear cells was similar among these groups (n = 7, each group). **D**) Cytokine levels in the skin culture supernatant were measured by flow cytometry. IL-1α and IL-1β were detected in conditioned medium from the skin culture of normal control mice and KIL-18Tg(−) mice, but was significantly higher in medium from skin culture of KCASP1Tg mice (n = 7, each group). **E**) Mouse adipose cells cultured in regular medium contained abundant lipid drops on day 14. The addition of supernatant from normal skin culture revealed a decrease in the number of plump adipocytes containing lipids as stained with oil red O, which was reversed by supplementing with supernatant from KCASP1Tg mice skin culture. The pretreatment of KCASP1Tg mice skin culture medium with anti-IL-1α or anti-IL-1β neutralizing antibodies partially ameliorated the inhibitory effects on adipose cells, which were almost abrogated by simultaneous treatment with both antibodies (n = 7, each group).

We treated KCASP1Tg mice with anti-IL-1α and/or IL-1β neutralizing antibodies when they were between 4 to 24 weeks old. Antibody treatment ameliorated body weight loss. Simultaneous injections of IL-1α and IL-1β antibodies produced additive effects (Fig. 2A). CT scans revealed that IL-1 antibody treatment prevented a decrease in the amount of somatic fat (Fig. S1B). The IL-1 antibodies also delayed the onset and progression of dermatitis, thereby supporting the idea that the skin pathology is accelerated by IL-1 [7]. Furthermore, to substantiate the pathogenic role of IL-1 in dermatitis and wasting syndrome, we treated normal healthy mice with recombinant IL-1α or IL-1β. Administration of either recombinant protein into wild-type mice caused a similar decrease in body weight within 10 weeks (Fig. 2B).

To study the underlying mechanisms by which sustained exposure of adipocytes to IL-1 induced emaciation, histopathological analysis of adipocytes was performed. While adipocytes from normal healthy mice appear as “plump nourished cells”, those from KCASP1Tg and KIL-18Tg(+) mice were small and round (Fig. 2C). Using a mouse embryonic fibroblast adipose cell line, we studied whether this atrophy of adipocyte was a direct effect of skin-derived cytokines. The skin culture supernatant from normal control mice and KIL-18Tg(−) mice contained low levels of IL-1α and IL-1β, whereas the skin culture supernatant from KCASP1Tg mice contained increased levels of those cytokines (Fig. 2D). The adipocytes cultured in normal media exhibited a plump shape containing abundant lipid particles (as stained with oil red O) (Fig. 2E). The addition of culture supernatant from normal skin resulted in a mild reduction of cytoplasmic lipid particles, thereby rendering the adipocytes less plump as a result of the cytokines released from the cultured normal skin. Furthermore, the addition of the KCASP1Tg mice skin culture supernatant induced severe reductions of lipid particles in adipocytes. The ability of KCASP1Tg mice skin supernatant to inhibit lipid particle growth was partially neutralized by either anti-IL-1α or IL-1β antibody. Indeed, this effect was abolished completely by simultaneous treatment with both antibodies (Fig. 2E).

### KCASP1Tg and KIL-18Tg(+) mice developed arteriosclerosis with impaired peripheral blood circulation and cardiomegaly

**Figure 3:**
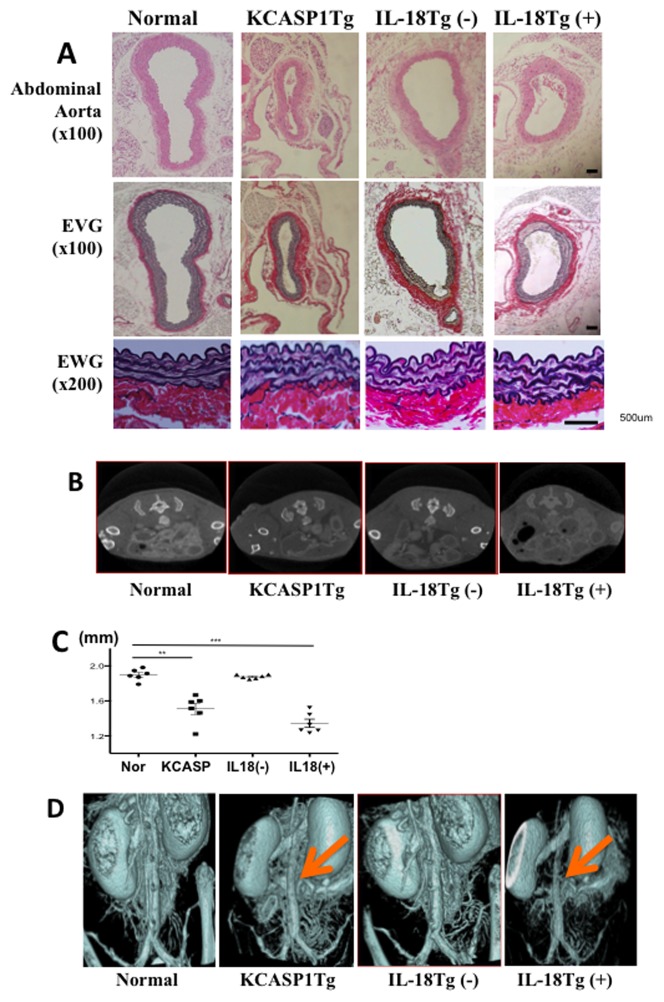
KCASP1Tg and KIL-18Tg(+) mice developed arteriosclerosis with impaired peripheral blood circulation. **A**) H and E staining of aorta sections revealed stenosis in 6-months old KCASP1Tg and 18-months old KIL-18Tg(+) mice. EVG staining revealed no significant changes in terms of periaortic lesions or elastic fibers. **B**) Enhanced CT scans revealed the presence of aortic stricture in KCASP1Tg and KIL-18Tg(+) mice. **C**) The aorta diameter in KCASP1Tg and KIL-18Tg(+) mice was decreased compared to normal control and KIL-18Tg(−) mice (n = 6, each group). **D**) Three-dimensional CT images showed the presence of aortic stenosis in KCASP1Tg and KIL-18Tg(+) mice.

**Figure 4:**
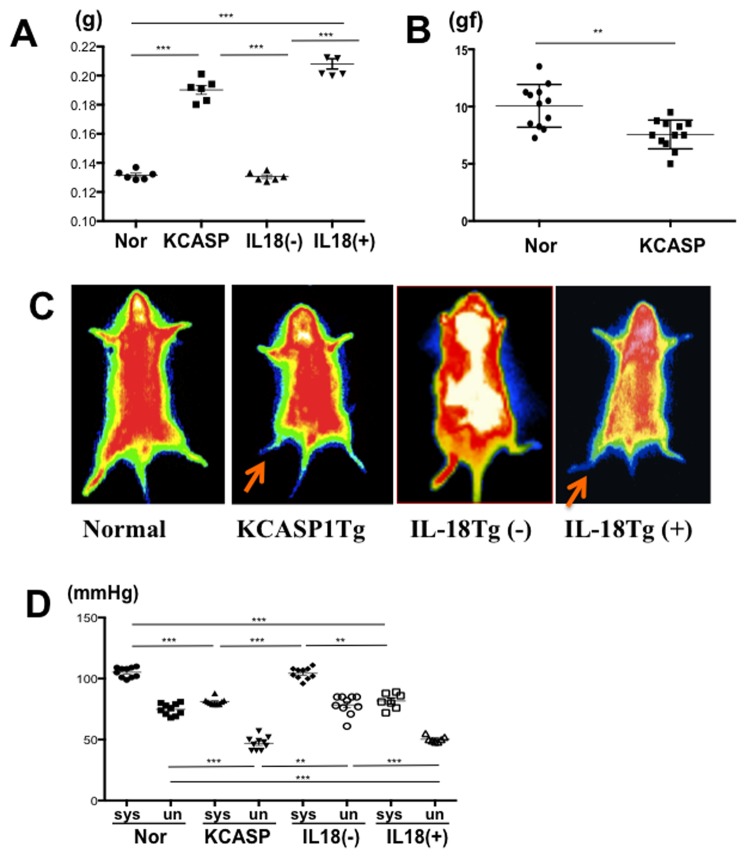
Cardiovascular findings in KCASP1Tg and KIL-18Tg(+). **A**) Six-months old KCASP1Tg mice had significantly heavier hearts compared to normal control and KIL-18Tg(−) mice. The heart weight of 18-months old KIL-18Tg(+) mice was also increased (n = 6, each group). **B**) The maximal tension produced in an 1 mm ring segment obtained from normal and KCASP1Tg mice is shown. The strength of the snapping point of the aorta ring from KCASP1Tg mice was significantly lower than in those from control mice (n = 12, each group). **C**) Thermography showed deterioration of peripheral blood circulation in the lower limbs and tail in 6-months old KCASP1Tg and 18-months old KIL-18Tg(+) mice. **D**) Both systolic and diastolic pressures were significantly lower in KCASP1Tg and KIL-18Tg(+) mice, (n = 10, each group) compared to control or KIL-18Tg(−) mice.

We next examined vessel structures. Aortic stenosis was observed in 6-months old KCASP1Tg mice and in 18-months old KIL-18Tg(+) mice (Fig. 3A). The smooth muscle (data not shown) and elastic fibers appeared normal in the vascular wall of stenotic aorta. We did not see any significant changes in the mRNA expression of the pro-fibrotic cytokine TGF-β1 and the anti-fibrotic cytokines IFN-γ and TNF-α in the aortas of controls or KCASP1Tg mice (data not shown). Atherosclerotic plaques were not detected. Enhanced CT scans revealed that the aorta diameter in KCASP1Tg and KIL-18Tg(+) mice was significantly reduced (Fig. 3B,C). Three-dimensional CT images of the abdominal aorta in KCASP1Tg and KIL-18Tg(+) mice clearly showed the presence of vascular stricture (Fig. 3D). In addition, these mice exhibited cardiomegaly with left ventricular hypertrophy (Fig. 4A). Ultrasonic echocardiography revealed no obvious left ventricular dysfunction in 6-months old KCASP1Tg mice (data not shown). Cardiomegaly was probably a secondary sign; it likely represents a compensatory response to aortic stenosis, and a consequence of reduced firmness and pliability of the aorta wall (Fig. 4B). Thermographic analysis revealed hampered circulation in lower limbs and tail of KCASP1Tg and KIL-18Tg (+) mice (Fig. 4C). Peripheral blood circulation further deteriorated after cold stimulation (data not shown), which is similar to that observed in patients with severe arteriosclerosis. The aortic stenosis was associated with impaired peripheral perfusion. The blood pressure measured at the tail was low during the systolic and diastolic phases in KCASP1Tg and KIL-18Tg(+) mice (Fig. 4D), thereby confirming impairment of peripheral circulation observed in the thermographic analysis.

### KCASP1Tg and KIL-18Tg(+) developed amyloidosis in the liver, kidney and spleen

**Figure 5:**
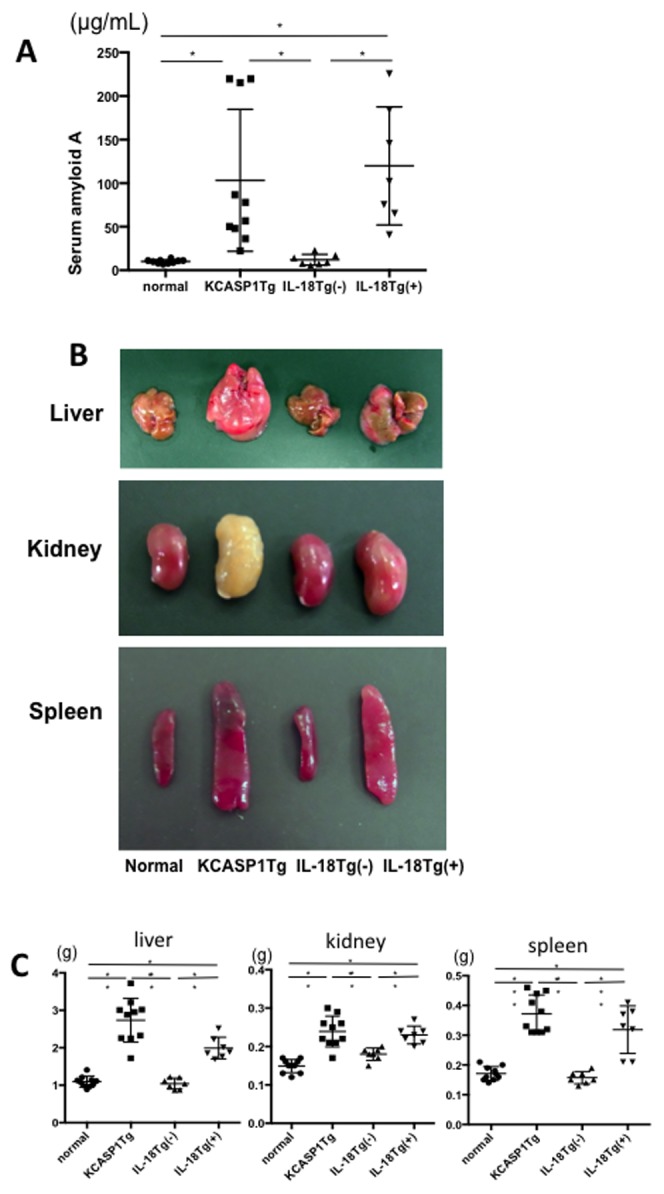
High serum amyloid A protein and organomegaly in KCASP1Tg and KIL-18Tg(+) mice. **A**) Serum amyloid A protein (SAA) levels were significantly higher in KCASP1Tg mice than in normal control and 6-months old KIL-18Tg(−) mice. Eighteen-months old KIL-18Tg(+) mice also showed elevated SAA concentration (n = at least 7). **B**) **C**) The liver, kidney, and spleen of both KCASP1Tg and KIL-18Tg(+) mice were significantly enlarged compared to control and KIL-18Tg(−) mice.

**Figure 6:**
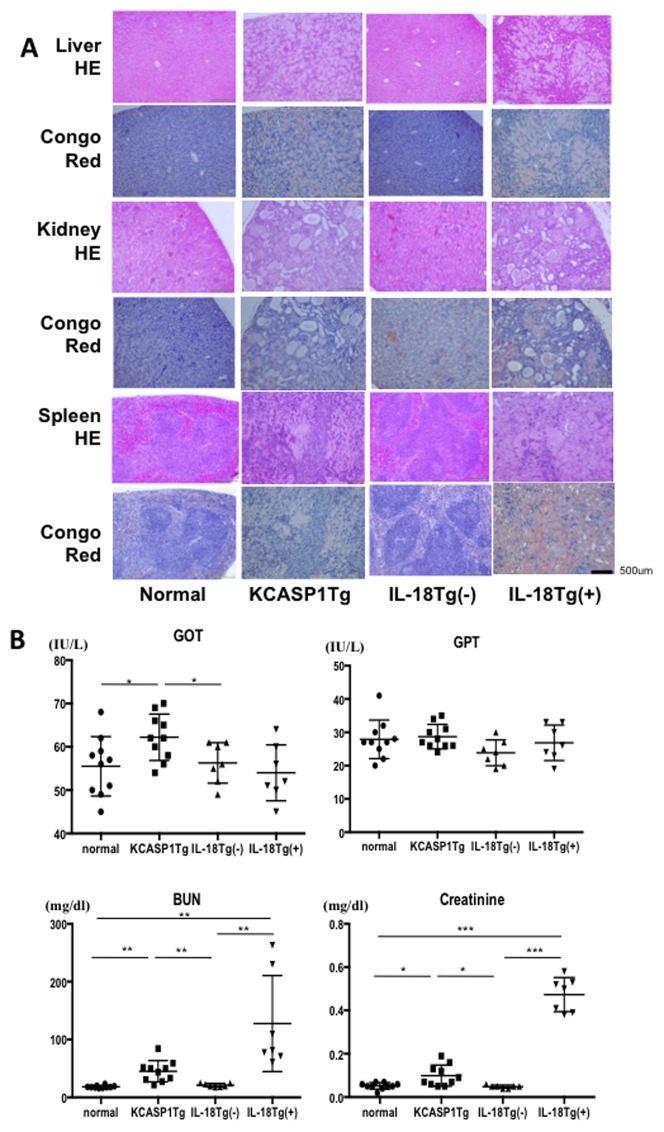
KCASP1Tg and KIL-18Tg(+) mice developed amyloidosis in the liver, kidney and spleen. **A**) Histological analyses showed loss of normal architecture: hepatocytes were replaced by dense deposits in the liver, the glomeruli and renal tubules were damaged in the kidney, and lymph-follicles were absent in the spleen. Dense amyloid deposition was detected in KCASP1Tg and KIL-18Tg(+) mice by Congo-red staining. **B**) KCASP1Tg mice showed mild liver and kidney dysfunction while renal function had significantly deteriorated in KIL-18Tg(+) mice (n = at least 7).

Chronic inflammation is often associated with amyloidosis. We investigated this by measuring the levels of serum amyloid A protein (SAA) and found significantly higher levels in KCASP1Tg and KIL-18Tg(+) mice than in control mice (Fig. 5A). The liver, kidney and spleen were hypertrophic in KCASP1Tg and KIL-18Tg(+) mice compared to normal controls (Fig. 5B,C). Histological examinations showed a dense amyloid deposition (Congo-red staining) and loss of normal architecture. Hepatocytes were replaced by dense deposits in the liver, and the damage of glomeruli and renal tubules were apparent in the kidneys, and lymph follicles were absent in the spleen (Fig. 6A). Liver damage as assessed by the serum level of transaminases was worse in KCASP1Tg mice than in control mice. Kidney function was mildly impaired in KCASP1Tg mice, and it was significantly deteriorated in KIL-18Tg(+) mice (Fig. 6B).

## Discussion

Several studies have shown the occurrence of systemic complications such as arteriosclerosis, cardiomyopathy, abnormal fat metabolism, renal sclerosis and systemic amyloidosis in severe inflammatory skin disorders. Here we demonstrated that sustained circulating level of IL-1 derived from severe skin inflammation causes weight loss, vascular sclerotic changes, cardiomegaly and severe systemic amyloidosis in multiple organs. Surprisingly, these pathologies were ameliorated by simultaneous treatment with anti-IL-1α and anti-IL-1β antibodies (Fig. 2A, Fig. S1 and S2). We hypothesized that mice generated by crossing KCASP1Tg with IL-1α/β double knock-out mice [18] (KCASP1Tg/IL-1α/β double knock-out mice) would have milder symptoms than KCASP1Tg mice due to overexpression of caspase-1 in a background lacking both IL-1α and IL-1β. Similar to the beneficial effects obtained with the antibody treatment, KCASP1Tg/IL-1α/β double knock-out mice also showed an improvement in the pathological phenotypes described here (Fig. S1 and S2). Although the number of KCASP1Tg/IL-1α/β double knockout mice is few due to the difficulty of mating, the significance between KCASP1Tg and KCASP1Tg/IL-1α/β double knockout mice reached the significance. Amelioration of emaciation was not complete in the crossed mice, however, suggesting the involvement of other inflammatory cytokines in the mechanism of emaciation; this is currently under investigation in our laboratory.

Leakage of IL-1α and IL-1β from injured keratinocytes induces atrophy of fat tissue/adipocytes, resulting in decreased somatic and subcutaneous fat. IL-1 is involved in the pathogenesis of arteriosclerosis obliterans (ASO) and atherosclerosis [19], monocytes being the source of IL-1 in ASO. Atheroma and sclerosis are characteristic findings of ASO. In many animal models of ASO, hyperlipidemia occurs prior to atheroma formation. Interestingly, KCASP1Tg and KIL-18Tg(+) mice showed no atheroma plaque formation, suggesting that, *in vivo*, IL-1 can induce arteriosclerosis without atheroma formation. Vascular atherosclerotic changes have also been reported in auto-inflammatory diseases including familial Mediterranean fever (FMF); in this disease, intermittent exposure to IL-1 [20] only leads to scant atheroma formation. The total and HDL cholesterol levels in FMF patients are usually low compared to levels observed in KCASP1Tg mice. The plasma IL-18 levels in KIL-18Tg mice began to rise at a young age (i.e., 4-weeks old) but within one-year of age these mice develop no vascular complications and have no elevation in the serum level of IL-1 suggesting a secondary role of IL-18 in the mechanism of sclerosis [7].

The results of the present study not only provide a valuable *in vivo* example for systemic pathologies caused by chronic and sustained high circulating level of IL-1, but also have important clinical connotations; for example, a massive release of IL-1 from the skin can occur if severe AD patients intensely and repeatedly scratch their itchy skin, further worsening skin damage. The results of the present study suggest the need to avoid scratching to improve the clinical control of inflammatory skin diseases including AD. Psoriasis is another chronic inflammatory disease of the skin characterized by an elevated circulating level of IL-1 induced by local overexpression of TNF-α or IL-12/23 p40; the increased circulating level of IL-1 has been shown to be a risk factor for cardiovascular disorders [21]. Biologics therapy targeting TNF-α or IL-12/23 p40 suppress skin lesions and by this mechanism reduces the risk of vascular complications [22]. In EB, The skin pathological phenotype of EB is caused by inflammation and by minor trauma as previous demonstrated [23], [24]. The IL-1β signaling is constitutively activated in EB keratinocytes [25], and the serum IL-1α and IL-1β levels are several-fold increased even in the mild type of EB [26]. Sustained skin disruption in EB patients can lead to aberrant skin secretion with high circulating level of IL-1 that may potentially cause emaciation, vascular disorder, systemic amyloidosis and other visceral pathologies. Recently, a hereditary disease with a new syndrome featuring severe dermatitis, multiple allergies and metabolic wasting syndrome (SAM syndrome) caused by homozygous mutations in DSG1 has been described [4]. DSG1 encodes desmoglein 1, a major constituent of desmosomes, which have a crucial role in maintaining epidermal integrity and barrier function. Mutations that cause SAM syndrome may lead to loss of cell-to-cell adhesion, induce calcium flux in keratinocytes with IL-1α release.

The results of the present study provide evidence supporting the morbid association of chronic and severe inflammation of the skin with cardiovascular complications. These novel observations may also explain the involvement of systemic organs in the hereditary inflammatory skin diseases such as EB, severe AD and SAM syndrome. Successful inhibition of complications in other systemic organs by the treatment with anti-IL-1 antibodies provides a tool for preventing the disease and to improve prognosis in this kind of patients. This is not limited to skin diseases but also to other disorders such as autoinflammatory disorders.

## Supporting Information

Figure S1 **Amelioration of clinical and pathological findings by neutralization and knockout of IL-1 in KCASP1Tg mice.** **A**) IL-1s neutralization with antibodies ameliorated the impaired peripheral circulation as demonstrated by thermography and aorta histopathological changes. IL-1α and IL-1β double knockout KCASP1Tg developed neither peripheral circulatory changes nor histopathological changes in aorta. **B**) Body fat ratio improved by IL-1 neutralization (n = 7) and by its deficiency (n = 4) in. KCASP1Tg mice. The data of normal and KCASP1Tg are taken from Figure 1C.
(TIF)

Figure S2 **Organ amyloidosis was ameliorated by neutralization and knockout of IL-1 in KCASP1Tg mice.** A) The H&E and Congo-red staining revealed that IL-1 neutralization or deficiency ameliorate amyloid deposition in the liver, kidney and spleen.
(TIF)
