# Supplementary figures and images for: Persistent Release of IL-1s from Skin Is Associated with Systemic Cardio-Vascular Disease, Emaciation and Systemic Amyloidosis: The Potential of Anti-IL-1 Therapy for Systemic Inflammatory Diseases
Source: PLoS One. 2014 Aug 13;9(8):e104479. doi: 10.1371/journal.pone.0104479 (PMC4131904; doi:10.1371/journal.pone.0104479)

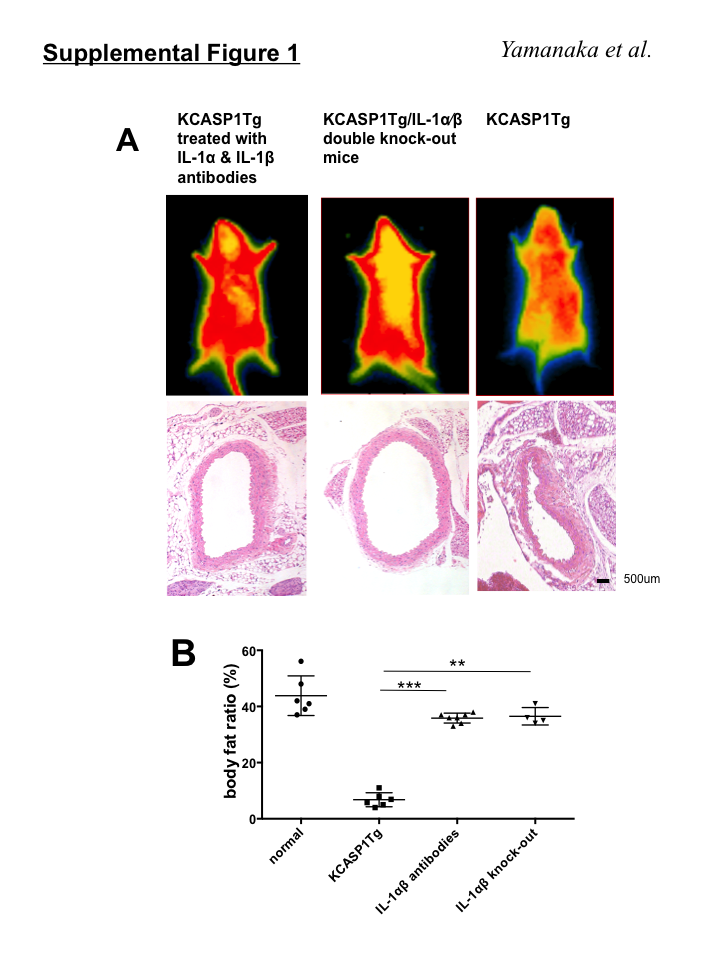

Supplement: Figure S1 — Amelioration of clinical and pathological findings by neutralization and knockout of IL-1 in KCASP1Tg mice. A) IL-1s neutralization with antibodies ameliorated the impaired peripheral circulation as demonstrated by thermography and aorta histopathological changes. IL-1α and IL-1β double knockout KCASP1Tg developed neither peripheral circulatory changes nor histopathological changes in aorta. B) Body fat ratio improved by IL-1 neutralization (n = 7) and by its deficiency (n = 4) in. KCASP1Tg mice. The data of normal and KCASP1Tg are taken from Figure 1C. (TIF) [file pone.0104479.s001.tif]

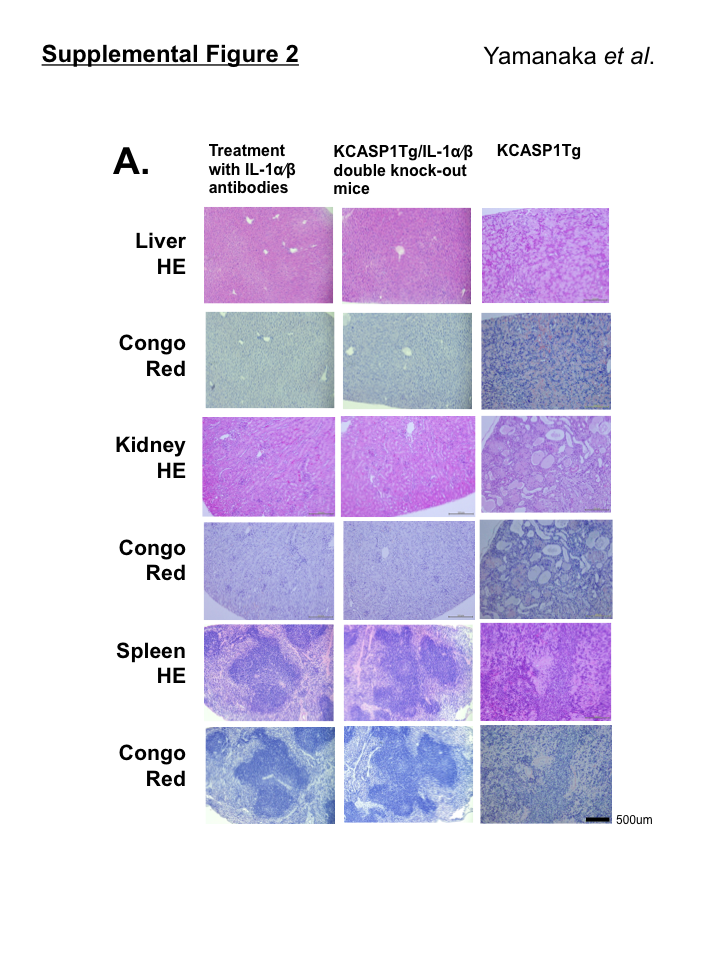

Supplement: Figure S2 — Organ amyloidosis was ameliorated by neutralization and knockout of IL-1 in KCASP1Tg mice. A) The H&E and Congo-red staining revealed that IL-1 neutralization or deficiency ameliorate amyloid deposition in the liver, kidney and spleen. (TIF) [file pone.0104479.s002.tif]
